# Systematic identification of transcriptional and post-transcriptional regulations in human respiratory epithelial cells during influenza A virus infection

**DOI:** 10.1186/1471-2105-15-336

**Published:** 2014-10-04

**Authors:** Zhi-Ping Liu, Hulin Wu, Jian Zhu, Hongyu Miao

**Affiliations:** Department of Biomedical Engineering, School of Control Science and Engineering, Shandong University, Jinan, Shandong 250061 China; Department of Biostatistics and Computational Biology, University of Rochester, Rochester, NY 14642 USA; Department of Microbiology and Immunology, University of Rochester, Rochester, NY 14642 USA

**Keywords:** Influenza virus infection, Regulatory network in epithelial cells, Dimension reduction, Dynamic Bayesian network, Constrained LASSO

## Abstract

**Background:**

Respiratory epithelial cells are the primary target of influenza virus infection in human. However, the molecular mechanisms of airway epithelial cell responses to viral infection are not fully understood. Revealing genome-wide transcriptional and post-transcriptional regulatory relationships can further advance our understanding of this problem, which motivates the development of novel and more efficient computational methods to simultaneously infer the transcriptional and post-transcriptional regulatory networks.

**Results:**

Here we propose a novel framework named SITPR to investigate the interactions among transcription factors (TFs), microRNAs (miRNAs) and target genes. Briefly, a background regulatory network on a genome-wide scale (~23,000 nodes and ~370,000 potential interactions) is constructed from curated knowledge and algorithm predictions, to which the identification of transcriptional and post-transcriptional regulatory relationships is anchored. To reduce the dimension of the associated computing problem down to an affordable size, several topological and data-based approaches are used. Furthermore, we propose the constrained LASSO formulation and combine it with the dynamic Bayesian network (DBN) model to identify the activated regulatory relationships from time-course expression data. Our simulation studies on networks of different sizes suggest that the proposed framework can effectively determine the genuine regulations among TFs, miRNAs and target genes; also, we compare SITPR with several selected state-of-the-art algorithms to further evaluate its performance. By applying the SITPR framework to mRNA and miRNA expression data generated from human lung epithelial A549 cells in response to A/Mexico/InDRE4487/2009 (H1N1) virus infection, we are able to detect the activated transcriptional and post-transcriptional regulatory relationships as well as the significant regulatory motifs.

**Conclusion:**

Compared with other representative state-of-the-art algorithms, the proposed SITPR framework can more effectively identify the activated transcriptional and post-transcriptional regulations simultaneously from a given background network. The idea of SITPR is generally applicable to the analysis of gene regulatory networks in human cells. The results obtained for human respiratory epithelial cells suggest the importance of the transcriptional, post-transcriptional regulations as well as their synergies in the innate immune responses against IAV infection.

**Electronic supplementary material:**

The online version of this article (doi:10.1186/1471-2105-15-336) contains supplementary material, which is available to authorized users.

## Background

Seasonal and pandemic influenza A virus (IAV) continues to be a public health threat and to exert a large economic burden worldwide [1]. IAV RNA segments that encode the hemagglutinin (HA) and neuraminidase (NA) proteins can undergo mutation (antigenic drift) or reassortment (antigenic shift), resulting in new viral strains that humans may lack the heterologous immunity against (e.g., the pandemic H1N1 2009 [2]). In such circumstances, the cell-mediated and humoral immune responses are primary, and a better understanding of the molecular mechanisms of immune responses to IAV infection thus becomes necessary to the development of more effective prevention and treatment strategies. Particularly, the regulations of cell gene expression are of significant importance due to their critical roles in shaping the magnitude and timing of immune responses via the promotion or suppression of the production of various proteins and RNAs (e.g., cytokines [3, 4] and miRNAs [5]). Since different cell populations with distinct phenotypes and functions are expected to have different gene regulation profiles, it is desirable to characterize the gene regulations for each cell population as in [6, 7]. In this study, the respiratory epithelial cells are of primary interest because they are the first barrier and the main target of IAV infection in human [8]. A number of previous studies have measured and analyzed the gene expression profiles in respiratory epithelial cells during IAV infection [9–12]; however, only a few studies investigated the gene expression regulators like transcription factors [13] or miRNAs [14]. Several studies in other biological disciplines show that TFs and miRNAs cooperatively interact with each other and both of them should be considered in the regulatory network model [15, 16]. To our best knowledge, simultaneous identification of both the transcriptional (TF) and post-transcriptional (miRNA) regulations on a genome-wide scale in human respiratory epithelial cells post IAV infection has not been sufficiently addressed.

A number of systems biology approaches have been developed to understand the transcriptional or post-transcriptional regulations [17–19], including several studies particularly for influenza H1N1 virus infection [13, 20, 21]. For example, Butte and Kohane [22] considered the pair-wise mutual information to screen the gene data associations. Friedman et al. [23] employed the Bayesian network model to capture the conditional independencies between genes. Yeung et al. [24] used the singular value decomposition (SVD) and the robust regression for network reverse engineering, given the sparsity of real large-scale networks. Zhang and Horvath [25] proposed the weighted gene co-expression network analysis, which assigns a continuous connection weight between 0 and 1 to each gene pair. Basso et al. [6] combined the mutual information with the data processing inequality theory to analyze the gene expression profiles in human B cells. Ordinary differential equation models have also been considered in the previous studies [26], in which the computing efficiency issues were addressed for high-dimensional dynamic systems. However, as evaluated in [27, 28], the accuracy of these existing methods are not satisfying, and the reliability of network reverse engineering from gene expression data needs to be further improved. More importantly, two key issues are not sufficiently addressed in the previous studies: first, while both TFs and miRNAs are the major regulators in controlling gene expression [19, 29], none of the methods mentioned above simultaneously considered both transcriptional and post-transcriptional regulations such that the inferred interactions could be biased; second, most of the existing approaches are purely data-driven and thus the results could have a high false positive rate [30–32].

In this study, we propose a new framework called SITPR, which stands for Systematic Identification of Transcriptional and Post-transcriptional Regulations, to identify the regulatory relationships by exploiting both curated knowledge and time-course expression data of mRNAs and miRNAs. For this purpose, a background regulatory network is constructed on a genome-wide scale by collecting the experimentally-observed interactions in literature or public databases [33, 34] as well as the potential regulatory interactions predicted by representative algorithms for quantifying the sequence-selective binding feasibility between TFs/miRNAs and genes [18, 35, 36]. Since this background network is not cell type or disease specific, only part of the interactions in this background will be activated in respiratory epithelial cells in response to influenza infection. To identify these activated regulatory interactions, we first employ several dimension reduction approaches, including the community detection algorithm based on network modularity [37], the time delay detection method [38, 39], the functional principal component analysis (fPCA) for screening differentially expressed genes [40], and the maximum information coefficient (MIC) [41]. After dividing the large network into much smaller modules, we propose the constrained LASSO method and combine it with the dynamical Bayesian network (DBN) model to detect the activated regulatory relationships from time-course expression data within each module. Our simulation studies suggest that the proposed framework can achieve satisfying performance in terms of sensitivity and specificity for networks of different sizes. We then apply the framework to the real experiment data and analyze the topological and functional features of the regulatory network in respiratory epithelial cells in response to H1N1 influenza virus infection.

## Results and discussion

### Background regulatory network obtained for human

Instead of inferring regulatory network only from expression profiling data, we first build a background regulatory network for human (see Additional file 1: Text S1 and Methods), and then identify the activated regulatory relationships during IAV infection using the mRNA and miRNA time-course expression datasets. We collect the documented regulatory relationships among TFs, miRNAs, and genes in various databases and literature, and also incorporate the potential regulatory relationships between regulators and targets predicted according to the sequence motifs of TFBSs (see Methods). For instance, we retrieve information from the ENCODE project [42] (Text S1) so the majority of the regulators and targets in ENCODE (102 out of 119 TFs, 723 out of 736 miRNAs and 13,607 out of 15,131 target genes) are included in our study. The resulted background regulatory network for human contains 23,079 nodes and 369,277 edges, consisting of 1,456 TFs, 1,904 miRNAs and 19,719 target genes. Among all these edges, 55.6% are experimentally validated interactions, and the rest are predicted regulatory relationships. Note that the regulatory interactions in the constructed background network are not limited to particular cell types or disease conditions; therefore, only part of these interactions will be activated under specific conditions (e.g., in respiratory epithelial cells infected by IAV).

For verification purpose, we calculate several statistical measures of the background regulatory network (Additional file 1: Table TS4), which clearly suggest that the obtained background network is different from a random network. More specifically, the clustering coefficient of our background network is 0.117, which is much higher than that of a random network of a comparable size (~ 1.5 × 10^- 5^) [43]. Also, the node degrees of our background network are found to satisfy the power-law distribution (Additional file 1: Figure TS3). Fitting the power law *y* = *α* ⋅ *x*^- *γ*^, where *y* denotes the number of nodes and *x* denotes the node degree, we obtain *γ* = 2.126, which is between 2 and 3 and thus suggests our background network is scale-free. This value also indicates that our background network is not random [43, 44].

The constructed background network is a high-dimensional network. To make the computing cost affordable, we employ several topology-based and data-based dimension reduction methods to divide the background network into smaller modules. We then identify the activated interactions within each module by fitting the dynamic Bayesian network model to the time-course expression data. To control the false positive rate, we also introduce the novel constrained LASSO formulation into the model fitting procedure.

### Algorithm performance evaluation and comparison

Before we apply the SITPR framework to real data, the algorithm performance should be evaluated and compared using networks of different sizes. More specifically, the network size in terms of total node number is 10, 50 or 100, which is chosen to be comparable to the node numbers of the real network modules obtained in this study (ranging from 2 to ~200). We use linear ordinary differential equations (ODEs) to match the structure of a given network and generate the simulated time-course expression data. To be consistent with the real data used in this study, simulated data are generated at six time points (*t* = 0, …, 5) with six replicates at each time point. Gaussian white noises with a standard deviation of 10% of the data mean are added to all the data points. Six commonly-used criteria are chosen to evaluate the algorithm performance, including sensitivity (SN), specificity (SP), accuracy (ACC), F-measure, Matthews correlation coefficient (MCC), and the Area Under ROC Curve (AUC),
1

where TP, FN, FP, and TN are the numbers of true positive, false negative, false positive, and true negative, respectively; also, AUC does not have a closed-form expression and is thus not given in Eq. (1).

**Figure 1:**
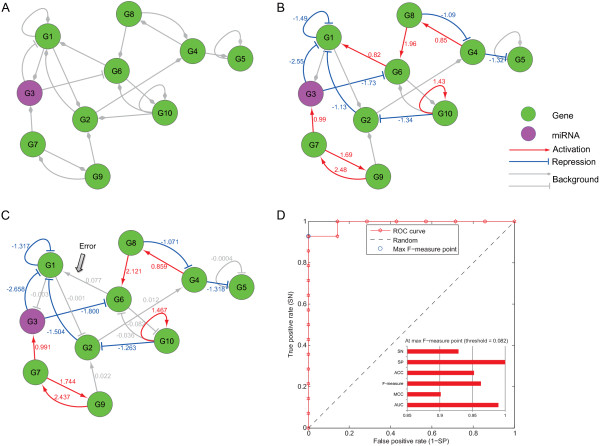
**Illustration of the performance evaluation of SITPR using an example regulatory network. (A)** The background regulatory network with 10 nodes. 'G3’ denotes a miRNA and is labeled in magenta. **(B)** The activated regulatory relationships (edges in color). The numbers next to the edges are the regulatory strengths. **(C)** An example of the inferred activated regulatory network using SITPR. **(D)** The ROC curve and the six performance evaluations at the maximum F-measure point.

For illustration purpose, we show a toy example of the regulatory system that consists of only 10 genes, which has a background regulatory network structure (in gray) as in Figure 1(A) and a set of activated regulatory relationships (in color) as in Figure 1(B). Furthermore, the miRNA is the G3 node labeled in magenta, and the true regulation coefficients are explicitly labeled next to each edge in Figure 1(B). The inferred regulatory relationships and coefficients from a randomly-chosen simulated dataset are shown in Figure 1(C), and the corresponding evaluation criterion values together with the ROC curve are plotted in Figure 1(D). Based on 10 simulation runs, we calculate the six criteria and the associated standard deviations. For this toy case, the proposed framework achieves a SN of 0.886 ± 0.090, SP of 0.971 ± 0.069, F-measure of 0.925 ± 0.070, MCC of 0.875 ± 0.144, ACC of 0.943 ± 0.667, and AUC of 0.966 ± 0.069 (also see Table 1).

**Table 1:** **Performance evaluation and comparison of SITPR, PCC, MI, CLR, ARACNE, GENIE3 and TIGRESS for networks of three different sizes**

Method	Node Size	SN	SP	ACC	F-measure	MCC	AUC
SITPR	10	0.886 ± 0.090	0.971 ± 0.069	0.943 ± 0.667	0.925 ± 0.070	0.875 ± 0.144	0.966 ± 0.069
	50	0.775 ± 0.089	0.635 ± 0.088	0.670 ± 0.054	0.690 ± 0.039	0.358 ± 0.062	0.703 ± 0.037
	100	0.690 ± 0.080	0.478 ± 0.101	0.530 ± 0.079	0.557 ± 0.080	0.147 ± 0.110	0.573 ± 0.071
PCC	10	0.400 ± 0.241	0.486 ± 0.125	0.457 ± 0.133	0.398 ± 0.204	-0.112 ± 0.293	0.596 ± 0.082
	50	0.495 ± 0.157	0.453 ± 0.167	0.463 ± 0.124	0.444 ± 0.105	-0.043 ± 0.189	0.557 ± 0.038
	100	0.534 ± 0.084	0.526 ± 0.057	0.528 ± 0.045	0.525 ± 0.050	0.052 ± 0.045	0.517 ± 0.027
MI	10	0.243 ± 0.243	0.679 ± 0.097	0.533 ± 0.092	0.293 ± 0.256	-0.101 ± 0.255	0.567 ± 0.062
	50	0.590 ± 0.145	0.527 ± 0.106	0.543 ± 0.065	0.542 ± 0.036	0.103 ± 0.072	0.574 ± 0.027
	100	0.539 ± 0.063	0.569 ± 0.079	0.561 ± 0.050	0.547 ± 0.030	0.095 ± 0.052	0.568 ± 0.024
CLR	10	0.400 ± 0.148	0.543 ± 0.096	0.495 ± 0.068	0.441 ± 0.081	-0.055 ± 0.143	0.540 ± 0.059
	50	0.565 ± 0.133	0.510 ± 0.090	0.523 ± 0.061	0.520 ± 0.072	0.064 ± 0.107	0.539 ± 0.066
	100	0.568 ± 0.130	0.543 ± 0.102	0.549 ± 0.059	0.538 ± 0.044	0.098 ± 0.084	0.550 ± 0.039
ARACNE	10	0.500 ± 0.168	0.643 ± 0.181	0.595 ± 0.117	0.532 ± 0.105	0.151 ± 0.213	0.577 ± 0.084
	50	0.620 ± 0.118	0.508 ± 0.119	0.535 ± 0.067	0.539 ± 0.0275	0.114 ± 0.062	0.547 ± 0.043
	100	0.576 ± 0.112	0.525 ± 0.108	0.537 ± 0.062	0.531 ± 0.039	0.089 ± 0.062	0.554 ± 0.050
GENIE3	10	0.429 ± 0.252	0.521 ± 0.147	0.490 ± 0.163	0.443 ± 0.21	-0.050 ± 0.345	0.609 ± 0.057
	50	0.565 ± 0.116	0.444 ± 0.228	0.473 ± 0.157	0.444 ± 0.162	-0.007 ± 0.187	0.529 ± 0.041
	100	0.512 ± 0.093	0.544 ± 0.062	0.536 ± 0.043	0.520 ± 0.050	0.049 ± 0.077	0.521 ± 0.033
TIGRESS	10	0.629 ± 0.168	0.586 ± 0.171	0.600 ± 0.127	0.583 ± 0.135	0.205 ± 0.233	0.586 ± 0.122
	50	0.490 ± 0.152	0.463 ± 0.105	0.470 ± 0.09	0.461 ± 0.095	-0.041 ± 0.167	0.536 ± 0.067
	100	0.554 ± 0.127	0.507 ± 0.083	0.519 ± 0.058	0.515 ± 0.070	0.053 ± 0.105	0.549 ± 0.033

For fairness of comparison when evaluating the performances, all the methods are assigned the same task (that is, identify the activated regulatory relationships for a given background network).

It is necessary to further evaluate the SITPR performance for background regulatory networks of a higher dimension. Since the number of nodes in the largest modules obtained by dividing the real background network (see Methods) is on the order of 100, we consider networks with 50 and 100 nodes. For this purpose, we adopt two network structures from the DREAM3 challenge [28] so both of them have the small-world property and an exponential degree distribution. The smaller structure has 50 nodes and 62 activated regulatory relationships, and the larger structure has 100 nodes and 125 activated regulatory relationships (see Additional file 2, in which 1 denotes a positive regulation and -1 the opposite). We randomly add more inactive edges to the two networks (denoted by 0 in Additional file 2: Table S1) so the smaller background network has 62 out of 82 edges are activated and the larger network has 125 out of 166 edges are activated. Based on such network structures, we use the linear ODE model again to generate simulated data at six time points with six replicates at each time point, and add 10% Gaussian white noise to each data point.

It is also necessary to compare the proposed framework with other representative or state-of-the-art reverse engineering approaches. For this purpose, we consider the Pearson’s correlation coefficient (PCC) method, the mutual information (MI) method [27, 28], and the best four algorithms in the DREAM challenge, including CLR [45], ARACNE [46], GENIE3 [47] and TIGRESS [48]. For fairness of comparison, the same task is assigned to all the methods under comparison: identify the activated regulatory relationships from a given background network. The parameter settings of the other methods under comparison are from the corresponding literature, and we make necessary modifications for these methods to take a background network as input.

In Table 1, we summarize the performance comparison results for three different network sizes (10, 50, and 100 nodes, respectively) based on 10 simulation runs. For the 10-node networks, the SITPR approach has a remarkable performance in comparison with the other six methods. For example, the AUC of SITPR is 0.966 ± 0.069, while the PCC, MI, CLR, ARACNE, GENIE3 and TIGRESS methods only achieve an AUC of 0.596 ± 0.082, 0.567 ± 0.062, 0.540 ± 0.059, 0.577 ± 0.084, 0.609 ± 0.057, 0.586 ± 0.122, respectively. For the 50-node network, the performance of the SITPR framework is again notably superior to those of the other methods, evidenced by the fact that all the six evaluation criteria of SITPR are the largest among all the approaches under comparison. For instance, the AUC of SITPR is 0.703 ± 0.037, while the MI and PCC methods have the second and the third best AUCs of 0.574 ± 0.027 and 0.557 ± 0.038, respectively. As the number of network nodes increase to 100, the performances of all these approaches decrease; however, the SITPR method still outperforms all the other methods in terms of, e.g., AUCs.

To assess the robustness of the SITPR method against data noise, we generate 100 simulated datasets for each of the three noise levels: let the standard deviation of the Gaussian white noise be 10%, 20% or 30% of the data mean. As suggested in Table 2, the performance of the SITPR method only marginally deceases as the noise level increases, so we conclude that its performance is robust against data noise. For example, for the three different noise levels, the AUCs of our method are 0.986 ± 0.045, 0.924 ± 0.133, and 0.889 ± 0.158, respectively.

**Table 2:** **Effects of the noise level and the total number of background edges on the performance of SITPR based on 100 simulation runs**

Noise	SN	SP	ACC	F-measure	MCC	AUC
10%	0.913 ± 0.086	0.994 ± 0.044	0.967 ± 0.057	0.950 ± 0.057	0.928 ± 0.093	0.986 ± 0.045
20%	0.867 ± 0.143	0.933 ± 0.141	0.911 ± 0.126	0.893 ± 0.131	0.816 ± 0.253	0.924 ± 0.133
30%	0.889 ± 0.182	0.886 ± 0.174	0.887 ± 0.155	0.876 ± 0.171	0.775 ± 0.304	0.889 ± 0.158
Background edge number	SN	SP	ACC	F-measure	MCC	AUC
40	0.884 ± 0.067	0.920 ± 0.071	0.897 ± 0.059	0.900 ± 0.059	0.787 ± 0.120	0.940 ± 0.060
80	0.854 ± 0.108	0.854 ± 0.104	0.854 ± 0.103	0.852 ± 0.100	0.629 ± 0.192	0.893 ± 0.095
100	0.829 ± 0.112	0.770 ± 0.093	0.820 ± 0.095	0.792 ± 0.083	0.521 ± 0.152	0.811 ± 0.071

We further evaluate the effects of the total number of edges in the background network on the SITPR performance. In Figure 1, we consider a background regulatory network with 14 activated interactions out of a total of 21 background regulatory relationships. Here we increase the number of background regulatory relationships to 40, 80 and 100, while the number of activated interactions remains at 14. As shown in Table 2, when the number of background regulatory relationships increases, the performance of our approach deceases marginally. Specifically, the AUCs of the SITPR method are 0.940 ± 0.060, 0.893 ± 0.095 and 0.811 ± 0.071 for 40, 80 and 100 background regulatory relationships, respectively.

In summary, the simulation study results provide direct evidences for the effectiveness and robustness of the proposed framework for systematically identifying activated transcriptional and post-transcriptional regulations from a given background network.

### Regulations in respiratory epithelial cells during IAV infection

We apply the SITPR framework to understand both the transcriptional and post-transcriptional regulations that are activated in human respiratory epithelial cells (A549 cell line) in response to H1N1 influenza virus infection. In short, from the constructed background regulatory network at the genome-wide scale, we obtain 303 functional modules (2 to ~200 nodes and 1 to ~300 edges in a module) after dimension reduction, and then identify the activated transcriptional and post-transcriptional regulations simultaneously in each module using the dynamic Bayesian network model together with the constrained LASSO formulation (see Methods).

Network motifs are known as the functional building blocks that control gene expressions [15, 49]. Therefore, after we determine the activated regulatory interactions from the background network, we further identify 10 types of 'TF-miRNA-gene’ co-regulation motifs using the FANDOM algorithm [50]. For convenience, the results are summarized in Table 3, in which the numbers of the 10 types of motifs in the background network and in the activated network are both listed. The detailed motif structures can be found in Additional file 1: Figure TS5. The statistical significance of each motif is also evaluated by calculating the associated Z-score. For this purpose, 1000 random networks are generated, and the Z-score is calculated as the difference between the motif occurrence in the real network and its average occurrence in the random networks, divided by the standard deviation of the occurrences in the random networks. We perform such calculations for both the background regulatory network and the activated regulatory network; and a motif with a Z-score greater than 2 is referred as enriched and statistically significant. For respiratory epithelial cells in response to H1N1 influenza virus infection, the ten type of motifs in the background regulatory network after data-based dimension reduction (see Methods) involve 20,310 regulatory relationships among 621 TFs, 642 miRNAs and 7,356 target genes, while nine types of these motifs can be found in the activated network, which involve 4,774 regulatory relationships among 420 TFs, 431 miRNAs and 3,773 target genes (Additional file 3). In these motifs, the numbers of the transcriptional and post-transcriptional regulations are of the same order of magnitude (e.g., 3,140 'TF-gene’ versus 1,449 'miRNA-gene’ regulations), which suggests that the importance of the post-transcriptional regulatory interactions may be comparable to that of the transcriptional regulatory relationships.

**Table 3:** **Ten co-regulation motifs and their occurrence frequencies in the background network and the activated network**

Motif ID	Network	Occurrence	Z-Score
M1	Background	1541	24.114
	Active	16	153.470
M2	Background	21317	8.412
	Active	209	2.356
M3	Background	45171	-3.933
	Active	109	-54.482
M4	Background	27987	3.685
	Active	153	1.053
M5	Background	187341	-1.152
	Active	1647	-20.769
M6	Background	2931	-1.091
	Active	-	-
M7	Background	20060	1.017
	Active	59	0.914
M8	Background	3226	-0.624
	Active	13	25.591
M9	Background	481293	-0.478
	Active	38	0.626
M10	Background	88102	-0.230
	Active	273	-24.802

Motifs are ordered according to their absolute Z-scores in the background regulatory network, which are calculated using FANMOD [50]. Motifs 'M1’, 'M2’, and 'M3’ are statistically significant in both the background and the activated regulatory networks (threshold of 2). 'M5’, 'M8’ and 'M10’ are not statistically significant in the background regulatory network, while they become significant in the activated regulatory network.

**Figure 2:**
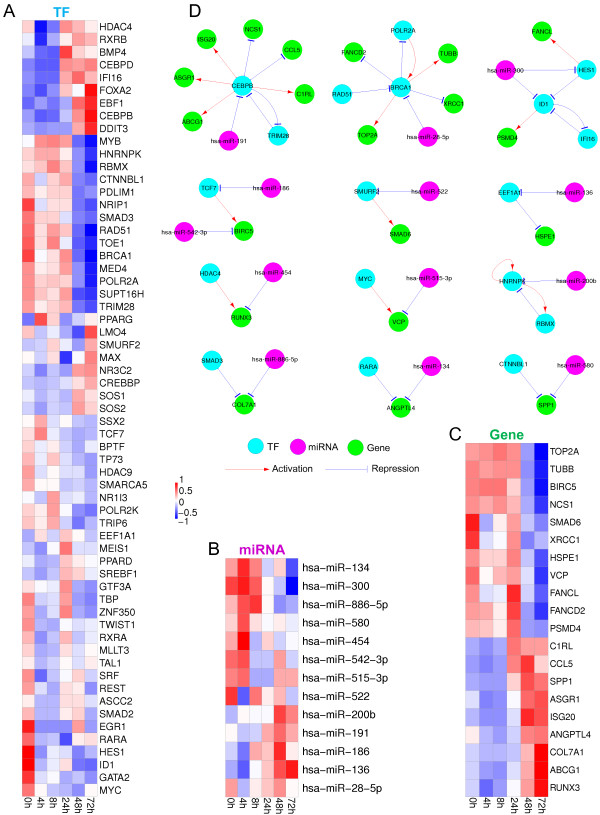
**Expression patterns of selected TFs (A), miRNAs (B) and target genes (C) in the combined co-regulation motifs (D).** The first motif cluster in **(D)** contains the motifs 'CEBPB’-'hsa-miR-191’-'CCL5’/'TRIM28’/'ISG20’/'ABCG1’ etc., and the second motif cluster contains 'BRCA1’-'hsa-miR-28-5p’-'TUBB’/'POLR2A’.

The motifs in the activated regulatory network (Table 3) are worth of further investigation to better understand the synergy of these motifs in gene expression regulation. We thus combine these co-regulation motifs into larger components if they are connected or overlap with each other. These components involved 61 TFs, 48 miRNAs and 127 genes (Additional file 4). Limited by space, we plot the expression profiles of a part of these TFs, miRNAs, and target genes in Figures 2(A), (B), and (C), respectively; the structures of a part of the components are sketched in Figure 2(D). A specific example is the component centered at 'CEBPB’ (CCAAT/Enhancer-Binding Protein Beta), which is an important TF involved in immune and inflammatory responses [51]. In this component (see the upper left plot in Figure 2(D)), 'TRIM28’ (tripartite motif containing 28, a co-factor for 'CEBPB’ involved in certain signal transduction pathways of glucocorticoids and inflammatory cytokines [52]) and 'CEBPB’ are found to regulate the expressions of each other, and their expression patterns shown in Figure 2(A) are consistent with this prediction. 'hsa-miR-191’ is identified as the primary miRNA that targets on the 'CEBPB’ mRNAs, through which the expressions of a number of important genes are indirectly co-regulated. More specifically, the 'CCL5’ (chemokine ligand 5) gene, which encodes the RANTES chemokine that plays an active role in leukocyte recruiting and NK cell activation and proliferation (together with IFN-γ) [53], is found to be the co-regulation target. The 'ISG20’ gene (interferon stimulated gene) is involved in the antiviral function of interferon against RNA viruses [51], and 'ABCG1’ (ATP-binding cassette subfamily G member 1) is involved in macrophage cholesterol and phospholipids transport, and is crucial for IAV replications and clearance responses of immune cells [51]. Both 'ISG20’ and 'ABCG1’ are also the target genes co-regulated by 'hsa-miR-191’ and 'CEBPB’. Such results provide further evidence for the importance of co-regulations in immune responses to influenza virus infection.

To gain a better view of the broad picture, we perform the functional enrichment analysis on each of the 303 modules using DAVID [54]. In Table 4, the enriched GO (gene ontology) biological processes [55] and the documented pathways in KEGG/REACTOME [33, 56] in 25 selected modules are listed for illustration purpose; for the complete analysis results, see Additional file 5. According to Table 4, the 'cell cycle’, 'cell proliferation’, 'positive regulation of programmed cell death’, and 'apoptosis’ are enriched, which suggests an active role of the cell-cycle related functions in response to IAV infection. In addition, a number of pathways (e.g., 'Influenza Infection’, 'Jak-STAT signaling pathway’, 'Wnt signaling pathway’, and 'MAPK signaling pathway’) are enriched in respiratory epithelial cells, with which a number of important immunological functions are associated (e.g., 'inflammatory response’, 'immune response’, 'I-kappaB kinase/NF-kappaB cascade’, 'cell activation during immune response’, and 'leukocyte activation during immune response’). For example, the interferon α/β receptor gene (IFNAR1) as well as the entire 'Jak-STAT signaling pathway’ in Module 106 are found enriched, which is consistent with the mechanism of the antiviral state development of lung epithelial cells conferred by Type I interferons [8, 57].

**Table 4:** **Functional terms of GO biological processes and KEGG/REACTOME pathways enriched in 25 selected regulatory modules (FDR < 0.05)**

Module	Term	Representative genes	***p***-value	FDR
Module103	GO:0008283 ~ cell proliferation	CREBBP	0.000353	0.00498
	GO:0006357 ~ regulation of transcription from RNA polymerase II promoter	CREBBP, EP300	0.003488	0.04824
Module106	GO:0019221 ~ cytokine-mediated signaling pathway	IRAK4	0.001318	0.0192
	hsa04630:Jak-STAT signaling pathway	IFNAR1, STAT2, IRF9	0.002251	0.0177
Module130	GO:0006915 ~ apoptosis	HSPE1	0.001585	0.01762
Module159	GO:0007249 ~ I-kappaB kinase/NF-kappaB cascade	IRF3, TRAF2, TICAM1, BCL3	3.58E-08	5.43E-07
	GO:0043068 ~ positive regulation of programmed cell death	TRAF2, TICAM1, BCL3	1.46E-07	2.21E-06
	GO:0002263 ~ cell activation during immune response	TICAM1, BCL3	2.23E-05	0.00034
	GO:0002366 ~ leukocyte activation during immune response	TICAM1, BCL3	2.23E-05	0.00034
	hsa04622:RIG-I-like receptor signaling pathway	IRF3, TRAF2, MAVS	2.27E-05	0.00021
	GO:0001819 ~ positive regulation of cytokine production	TRAF2, MAVS, TICAM1, BCL3	0.000347	0.00526
	GO:0051251 ~ positive regulation of lymphocyte activation	TRAF2, TICAM1	0.000433	0.00655
	GO:0045321 ~ leukocyte activation	TICAM1, BCL3	0.00047	0.00711
	GO:0006955 ~ immune response	MAVS, TICAM1, BCL3	0.000483	0.00731
	GO:0009615 ~ response to virus	IRF3, MAVS, TICAM1, BCL3	0.000609	0.0092
	REACT_6900:Signaling in Immune system	IRF3, TICAM1	0.001402	0.00804
Module171	hsa04310:Wnt signaling pathway	JUN, MAPK10, DVL2	0.000233	0.00246
	hsa04010:MAPK signaling pathway	JUN, MAPK10, MAPK1	0.001514	0.01594
	GO:0034097 ~ response to cytokine stimulus	JUN	0.00196	0.03023
	GO:0002237 ~ response to molecule of bacterial origin	JUN, MAPK1	0.002673	0.04102
Module173	GO:0006954 ~ inflammatory response	CEBPB, CCL5, F8, BDKRB1, ITGB2, HIF1A, TF, F3, C1RL, IL8	3.00E-05	0.00048
	GO:0009611 ~ response to wounding	CEBPB, CCL5, F8, BDKRB1, IGFBP1, ITGB2, NRG1, HIF1A, TF, F3, C1RL, IL8	5.28E-05	0.00085
	GO:0051240 ~ positive regulation of multicellular organismal process	CCL5, PTGS2, NRG1, HIF1A, MYLK2, TF, F3, IL27RA	0.000197	0.00316
	GO:0006952 ~ defense response	CEBPB, CCL5, F8, BDKRB1, ITGB2, HIF1A, TF, F3, IL27RA, C1RL, IL8	0.000835	0.01331
	GO:0032101 ~ regulation of response to external stimulus	CCL5, VEGFA, PTGS2, NT5E, F3, IL8	0.001131	0.01799
	REACT_604:Hemostasis	STX4, SLC7A7, F8, VEGFA, ITGB2, ALB, TF, F3	0.001131	0.00976
	GO:0002526 ~ acute inflammatory response	CEBPB, F8, TF, F3, C1RL	0.0014	0.02222
	GO:0043069 ~ negative regulation of programmed cell death	CEBPB, VEGFA, NRG1, ALB, KRT18, PPT1, PCSK6, F3	0.001937	0.03062
Module174	GO:0051726 ~ regulation of cell cycle	SMAD3, CDK4	1.10E-05	0.00016
	GO:0031328 ~ positive regulation of cellular biosynthetic process	SMAD3, CDK4	0.000612	0.00894
	GO:0006350 ~ transcription	SMAD3, ASH2L	0.000854	0.01245
Module175	GO:0000122 ~ negative regulation of transcription from RNA polymerase II promoter	CTNNB1,	0.002252	0.02972
Module178	GO:0051252 ~ regulation of RNA metabolic process	STAT1, UBE2I, HDAC3, PIAS2, DAXX, SP100	5.05E-05	0.00076
	REACT_11061:Signalling by NGF	HDAC3, AKT1	0.000413	0.00293
	GO:0007049 ~ cell cycle	DAXX, UBE2I, HDAC3, AKT1	0.0005	0.00753
	hsa04630:Jak-STAT signaling pathway	STAT1, AKT1, PIAS2	0.004991	0.04684
Module179	GO:0006974 ~ response to DNA damage stimulus	RAD54L, FANCI, XAB2, BCCIP, BRCA1, XRCC1, EEPD1, UPF1, RAD51, TOP2A, FANCD2, RAD54B	3.39E-08	4.91E-07
	REACT_216:DNA Repair	FANCI, XAB2, BRCA1, XRCC1, POLR2K, RAD51, POLR2A, FANCD2	1.64E-07	1.25E-06
	GO:0033554 ~ cellular response to stress	FANCI, RAD54L, XAB2, EEPD1, BRCA1, XRCC1, TOP2A, RAD54B, DHX9, BCCIP, UPF1, RAD51, FANCD2	2.71E-07	3.92E-06
	GO:0007049 ~ cell cycle	FANCI, RAD54L, KIF15, BRCA1, CHTF18, RCC1, RAD54B, CIT, BCCIP, UPF1, TUBB, RAD51, FANCD2	7.43E-06	0.00011
Module180	GO:0010604 ~ positive regulation of macromolecule metabolic process	NUP62	7.41E-05	0.0011
Module182	GO:0006303 ~ double-strand break repair via nonhomologous end joining	PRKDC	1.88E-06	2.83E-05
	GO:0045935 ~ positive regulation of nucleobase, nucleoside, nucleotide and nucleic acid metabolic process	ILF2, RELA, NFKB1, PRKDC	0.000934	0.01395
	GO:0002562 ~ somatic diversification of immune receptors via germline recombination within a single locus	PRKDC	0.001732	0.02573
Module184	GO:0022402 ~ cell cycle process	MLH1, MAP2K6	0.001302	0.01777
Module185	GO:0007243 ~ protein kinase cascade	SRC, IKBKB, NFKBIA	0.001885	0.02759
Module186	REACT_1538:Cell Cycle Checkpoints	MCM4, MCM3, MCM5, MCM7, MCM2	9.90E-23	7.84E-22
	GO:0006260 ~ DNA replication	MCM4, MCM3, MCM5, MCM7, MCM2	1.13E-21	1.51E-20
Module189	GO:0032268 ~ regulation of cellular protein metabolic process	CBS	0.00036	0.00532
Module190	GO:0008380 ~ RNA splicing	SFPQ	2.23E-06	3.19E-05
Module200	REACT_6185:HIV Infection	NUP188, NUP205	0.000592	0.00484
Module206	hsa04350:TGF-beta signaling pathway	SMURF2, SMAD6	0.001698	0.00872
Module241	GO:0006913 ~ nucleocytoplasmic transport	NXT1, RAN	0.001111	0.01645
Module244	REACT_12472:Regulatory RNA pathways	POLR2H, POLR2I, POLR2B, POLR2D, POLR2L	5.52E-10	3.65E-09
	REACT_6143:Pausing and recovery of Tat-mediated HIV-1 elongation	POLR2H, POLR2I, POLR2B, POLR2D, POLR2L	4.94E-09	3.27E-08
	REACT_6167:Influenza Infection	POLR2H, POLR2I, POLR2B, POLR2D, POLR2L	4.39E-06	2.91E-05
Module253	GO:0000398 ~ nuclear mRNA splicing, via spliceosome	HNRNPM	0.003042	0.04485
Module267	hsa04920:Adipocytokine signaling pathway	RXRB	8.48E-06	7.22E-05
Module280	GO:0006984 ~ ER-nuclear signaling pathway	PAK1, EIF2AK3	0.001649	0.02486
Module303	hsa04144:Endocytosis	VPS28	0.000182	0.00041

'Module’ refers to the module indices we gave. 'Term’ refers to the enriched GO terms (e.g. GO:0008283), KEGG pathways (e.g. hsa04630), and REACTOME pathways (e.g. REACT_6900). Certain genes in the modules are listed as 'Representative genes’. '*p*-value’ and 'FDR’ shows the statistical significance of the results.

**Figure 3:**
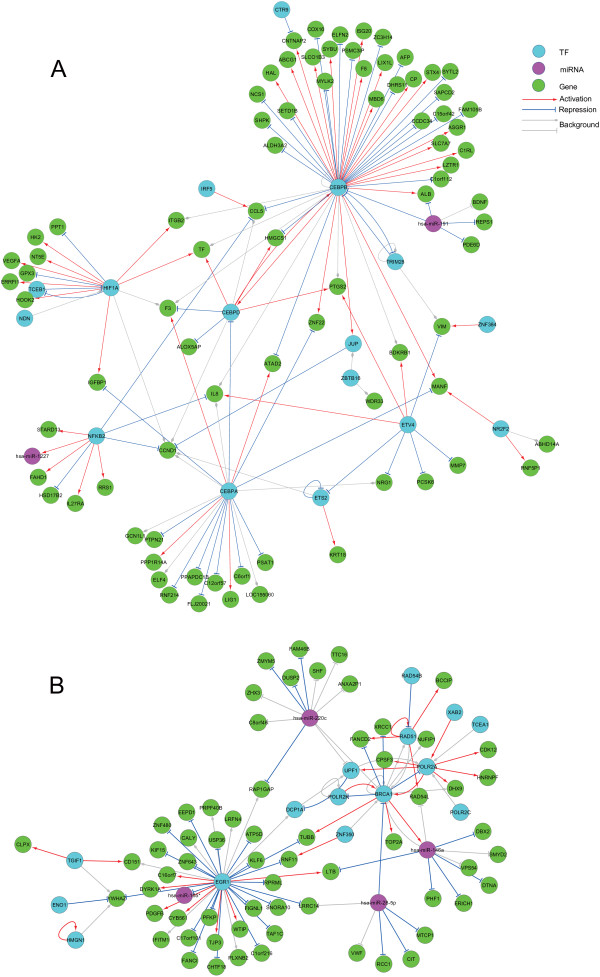
**The activated regulatory relationships in two example modules.** TFs, miRNAs and genes are in cyan, magenta and green, respectively. The up-regulation and down-regulation are labeled in red and blue, respectively. The background regulatory relationships which are not activated are in gray. The 'arrow’, 'T’ and 'diamond’ shapes of edge terminals denote to up-, down-, and undetermined- regulations, respectively. **(A)** Module173. This module contains the co-regulation motifs 'CEBPB’-'hsa-miR-191’-'CCL5’ and 'CEBPB’-'hsa-miR-191’-'ALB’/'ISG20’. It also contains some two-node regulatory motifs, e.g., 'ETS2’-'ETS2’, 'NFKB2’-'hsa-miR-1227’, 'IRF5’-'CCL5’ and 'hsa-miR-191’-'REPS1’. **(B)** Module179. This module contains the regulatory motifs 'BRCA1’-'has-miR-28-5p’-'TUBB’/'POLR2A’, “EGR1’-'hsa-miR-155*’, 'EGR1’-'hsa-miR-146a’-'LTB’ and 'BRCA1’-'hsa-miR-146a’-'PHF1’.

Figures 2(A) and (B) illustrate the activated regulatory relationships in two example modules (Modules 173 and 179) at a higher level of granularity, corresponding to the two components from the left of the first row sketched in Figure 2(D). For transcriptional regulations in Figure 3, 'CCL5’ is found to be regulated by 'IRF5’ (interferon regulatory factor 5) and 'NFκB2’ (nuclear factor kappa-B P100) besides 'CEBPB’. The induction of 'CCL5’ by 'IRF5’ and the regulatory roles of 'NFκB’ and 'CEBPB’ in 'CCL5’ expression have been reported in previous studies [53, 58, 59] , and are successfully identified by our method. In addition, the chemokine 'IL-8’ is found to be regulated by two TFs, 'NFκB2’ and 'ETV4’ (ETS translocation variant 4) [34, 53]. However, for such many-to-one regulatory relationships, it is unclear which TF is the major regulator and further experiments are thus needed to clarify this issue. Multiple post-transcriptional regulations can also be found in Figure 3. For example, 'hsa-miR-191’ is identified to suppress gene 'REPS1’ (RALBP1 associated Eps domain containing 1) in Figure 3(A), and 'hsa-miR-28-5p’ to suppress gene 'CIT’ (rho-interacting, serine/threonine kinase 21) in Figure 3(B). 'REPS1’ is involved in cytoskeletal signaling pathway and 'CIT’ encodes a serine/threonine protein kinase that functions in cell division and functions to promote efficient cytokinesis [51]. The repression of the two genes by miRNAs suggests a disorder of host factors caused by IAV infection via post-transcriptional regulations. Moreover, 'IFNGR2’ (interferon gamma receptor 2) suppression by 'hsa-miR-644’ and 'IFNG’ (interferon gamma) suppression by 'hsa-miR-26a’ are identified in Modules 107 and 287, respectively. This indicates the importance of miRNAs in innate immune responses against IAV infection by interfering IFN production and signaling [53, 60]. As an example of co-regulations, the 'hsa-miR-28-5p’-'BRCA1’-'TUBB’ regulation motif can be seen in Figure 3(B). 'TUBB’ (tubulin beta class I) is a major constituent of microtubules [51] in the REACTOME 'Cell cycle’ pathway [56], and is also a known human host proteins for IAV replication [21]. 'BRCA1’ is well known as a key mediator for repairing DNA damage by maintaining the genomic integrity [51]. A better understanding of the activated regulation among 'hsa-miR-28-5p’, 'BRCA1’ and 'TUBB’ could be important to designing the miRNA gene therapeutic trials targeting IAV host factors [21].

## Conclusions

In this study, we proposed a novel framework for systematic identification of transcriptional and post-transcriptional regulations (SITPR) using curated knowledge, sequence-based predictions and time-course expression data. Briefly, a comprehensive background regulatory network, which is not cell type- or disease-specific, was constructed at the genome-wide scale using information from ~20 databases and predictions from representative algorithms. After dividing the background network into smaller modules, we further proposed the constrained LASSO method and applied it with the dynamic Bayesian network model to each of the modules to determine the activated regulatory relationships based on the time-course expression data of mRNAs and miRNAs. The simulation studies clearly suggest the superiority of the proposed framework over other representative approaches in the context of identifying activated regulatory relationships from a given background network. The SITPR framework was then applied to real data to identify the activated regulatory relationships among TFs, miRNAs and target genes in human respiratory epithelial cells in response to H1N1 influenza virus infection.

Different from many existing methods for inferring gene regulatory network that are purely data-driven [17, 27, 28], we incorporated curated knowledge and utilized sequence information besides time-course gene expression data. Including curated knowledge can improve the accuracy of identifying genuine regulatory relationships [30, 32, 61], and the incorporation of predicted potential regulatory interactions among TFs, miRNAs and genes allows to discover novel regulatory relationships between regulators and targets. Furthermore, the way we constructed the background network (e.g., using predictions of sequence binding instead of associations between expression data [42, 62, 63]) and the use of the constrained LASSO help to control the false positive rate in identifying activated regulatory relationships, as suggested in [64, 65]. Also, since we considered the transcriptional and post-transcriptional regulations simultaneously, the identified activated regulatory relationships could be less biased [62, 63, 66, 67]. It should be mentioned that, limited by data availability and algorithm prediction accuracy, our background network built in this study is not thorough; however, the SITPR framework is flexible and allows us to update the background network whenever new knowledge is discovered or better prediction algorithms are devised.

Using the proposed framework, the transcription regulatory landscape was depicted for human respiratory epithelial cells infected by H1N1 influenza A virus. The transcriptional and post-transcriptional regulations were simultaneously identified and analyzed for airway epithelial cells in response to IAV infection. Besides pairwise interactions, we paid particular attention to regulation motifs and their synergistic effects on gene expressions. We identified ten regulation motifs using FANDOM and selectively presented and discussed some important motifs activated during IAV infection, which could suggest potential targets for influenza treatment or vaccination. It should be addressed that the regulation motifs we defined in this study are not only the functional building blocks but also the topological elements in a complex regulatory network. Identification of such motifs can provide the basis for advanced analyses such as differential network biology [68].

**Figure 4:**
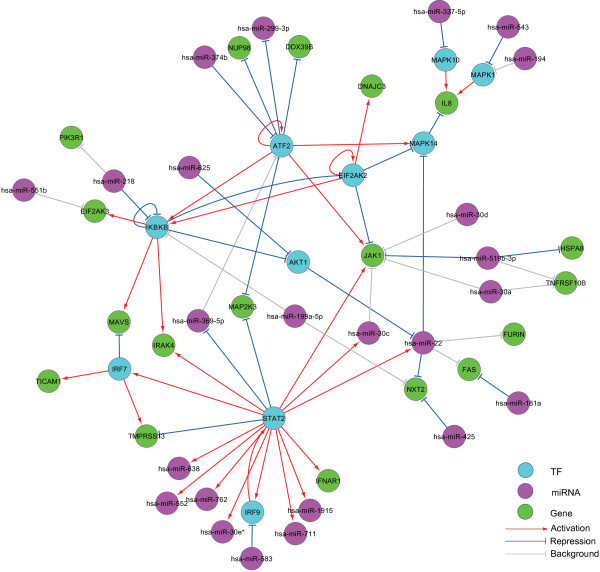
**The activated regulatory network in the KEGG IAV gene set.** The genes in the IAV pathway together with their neighbors from the background network were analyzed. The importance of miRNAs can be told from the co-regulation motifs, such as 'STAT2’-'hsa-miR-583’-'IRF9’, 'ATF2’-'hsa-miR-374b’-'JAK1’, 'IKBKB’-'hsa-miR-218’-'AKT1’, and 'MAPK1’-'hsa-miR-543’-'IL8’.

The SITPR is also applicable to a pre-defined gene set. For example, Figure 4 illustrates the activated regulatory relationships in the KEGG IAV pathway, in which we included the genes contained in this pathway as well as the neighboring miRNAs in our background network. One can tell from Figure 4 that 'STAT2’ (signal transducer and activator of transcription 2) directly regulates the expressions of 9 different miRNAs, 5 target genes and two other TFs, suggesting a highly influential role of 'STAT2’. In addition, we found that 'STAT2’, 'IRF9’ (interferon regulatory factor 9) and 'hsa-miR-583’ form a co-regulation motif. 'STAT2’, 'hsa-miR-519b-3p’ and 'JAK1’ (Janus kinase 1) also forms another co-regulation motif. 'STAT2’, 'JAK1’ and 'IRF9’ are known to be the key regulators in the 'Jak-STAT signaling pathway’, which plays a critical role in regulating immune responses [33]. The co-regulation motif 'MAPK1’-'hsa-miR-543’-'IL8’ was identified, which may indicate an important role of miRNA 'has-miR-543’ in controlling the production of the chemokine 'IL8’. In short, one obvious benefit of analyzing a pre-defined gene set is that the network dimension is significantly reduced such that SITPR can achieve a better accuracy, as suggested by our simulation studies. Loveday et al. [14], in which the time-course expression data used in this study were generated, also conducted a regulation study on a pre-defined gene set. However, they selected hundreds of differentially expressed genes and only focused on the post-transcriptional regulations between miRNAs and target genes. Also, the regulatory relationships identified in Loveday et al. [14] were primarily based on the baseline and the data on hour 8. Therefore, although there exist a few regulatory relationships identified in both our study and Loveday’s (e.g., 'hsa-miR-328’ and 'PSME3’), the majority of the regulatory interactions identified in the two studies are different due to all the reasons described above.

In summary, the background regulatory network constructed in this study provides a start point for the identification of condition-specific regulatory interactions. Together with the selected dimension reduction techniques, the SITPR framework is formed to achieve a better accuracy at an affordable computing cost. Also, for human respiratory epithelial cells in response to influenza A virus infection, the results generated using SITPR reveal a number of interesting activated regulatory interactions and provide useful clues for future experimental design and validation.

## Methods

### Expression data and experiments

The time-course expression data of mRNA and miRNA in human A549 cells infected with influenza H1N1 virus (A/Mexico/InDRE4487/2009) at a multiplicity of infection (MOI) of 0.1 were downloaded from NCBI GEO [69] (GSE36553 and GSE36461). The samples of mRNA and miRNA were hybridized on Illumina HumanHT-12 v3 BeadChips and Febit miRBase 14 Geniom miRNA Biochip, respectively. Data at six time points (0, 4, 8, 24, 48, and 72 hours post infection) were collected with six replicates at each time point [14]. However, one chip sample on hour 4 was found to be degraded [14], so only 35 samples were usable for analysis. More details of the experiment and raw data preprocessing can be found in the original manuscript [14]. We mapped the probeset IDs to NCBI official gene symbols using the GEO annotation file. When multiple probesets were mapped to the same gene, the probeset with the maximum inter quartile expression range was selected.

### Construction of the background regulatory network

**Figure 5:**
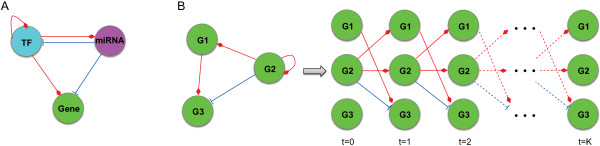
**Illustration of regulatory relationships and the dynamic Bayesian network (DBN) model. (A)** Five types of regulatory relationships among TF, miRNA and target gene. **(B)** Example of a DBN model for a 3-node network.

TFs and miRNAs are the major transcriptional and post-transcriptional regulators, respectively [18, 19]. Simultaneously considering the interplays among TFs, miRNAs and target genes could result in a more accurate identification of regulatory relationships [62]. Therefore, five types of regulatory relationships need to be accommodated in the background network as illustrated in Figure 5(A): 'TF-gene’, 'miRNA-gene’, 'TF-miRNA’, 'miRNA-TF’ and 'TF-TF’ self-regulation. For this purpose, we collected a large number of documented regulatory relationships from about 20 public databases and predicted the potential regulations among TFs, miRNAs and target genes by scanning the sequence binding motifs. It should be addressed that the background regulatory network is not cell type or disease specific since it is constructed at the genome-wide scale. Also, the background network allows the discovery of novel regulations by including potential regulations. Instead of associations in gene expression data, the potential regulations are predicted by matching the sequence binding motifs of targets, which is helpful to reduce the false positive rate (FPR) in the inferred regulations [70].

Specifically, human TFs were compiled from FANTOM [71], TRANSFAC [35] and JASPAR [36]; human miRNAs were downloaded from miRBase [72]; and human genes and annotations were from NCBI GenBank and RefSeq [73]. For convenience, all the human TFs and genes were denoted using their NCBI official gene symbols. For the 'TF-gene’ and 'TF-TF’ regulations, we downloaded the curated regulations from TRED [34] and KEGG [33], and the regulatory DNA elements of transcriptional factor binding sites (TFBS) were extracted from TRANSFAC [35] and JASPAR [36]. For binding motifs in TRANSFAC, we identified the putative binding regions of TFs in human genome using the tables tfbsConsSites and tfbsConsFactors from UCSC Genome Browser [74], as suggested by the ENCODE project [42]. When mapping to the human genome (UCSC h19 human genome assembly), the binding regions were searched within the promoter region from 5-kb upstream to 1-kb downstream of the transcription start site (TSS) annotated by RefSeq. We used the default *Z*-score of 2.33 to control the type I error in binding region prediction. For JASPAR TFBS annotation, we used the MotifFeatures and AnnotatedFeatures tables from Ensembl [75] and extracted the TFs and their binding sites regions in the human genome. Again, we mapped the binding sites to human genome and identified the regulatory relationships between TFs and their potential targets. For completeness, we also extracted the TF and their target proteins from human protein-protein interaction data in HPRD [76] and KEGG [33], and mapped such interactions to the 'TF-gene’ and 'TF-TF’ regulations, which allows a more thorough and systematic exploration of the regulatory interactions as suggested in previous studies [62]. For the 'TF-miRNA’ regulations, the previously-reported TF-miRNA interactions were downloaded from TransmiR [77]. We used a procedure similar to that for the 'TF-gene’ and 'TF-TF’ regulations to identify the regulations between TFs and miRNA’s corresponding genes. For the 'miRNA-gene’ and 'miRNA-TF’ regulations, the curated miRNA-gene interactions are available in public databases such as Tarbase [78], miRecords [79] and miRTarBase [80]. There are also numerous computing methods for predicting miRNA targets, such as TargetScan [18] , miRanda [81], PicTar [82], microT [83] and MicroCosm [72]. We downloaded the experimentally-confirmed miRNA-gene interactions from the three pubic databases, and the potential interactions were kept only if they were predicted by at least two of the five algorithms mentioned above.

Finally, we built a background regulatory network for human with 23,079 nodes and 369,277 edges, consisting of 1,456 TFs, 1,904 miRNAs and 19,719 target genes. The statistics and more details of the background network are available in Additional file 1. For the real data we used in the Results Section, 37,586 mRNAs and 904 miRNAs were measured in the experiments. The overlap between the background network and the experiment data contains 18,964 nodes (1,441 TFs, 881 miRNAs, and 16,642 target genes) and 335,963 interactions. All the data and codes can be accessed at our website http://doc.aporc.org/wiki/SITPR.

## Dimension reduction

Although the number of edges in the background network is significantly smaller than that of a fully connected network, the computing cost is still prohibitively high if we directly use the entire background to infer the activated regulatory relationships. We thus considered several data-based and topological feature based methods for dimension reduction.

First, we only kept the differentially expressed genes during viral infection, which were identified at a significance level of 0.05 using our functional PCA approach particularly designed for time-course data [40]. Second, we filtered out the regulatory pairs if the initial change in the regulator gene expression lags behind that in the target gene expression [38]. For this purpose, we clustered the time-course expression data of mRNAs and miRNAs using STEM [39] and then determined the time of initial change in gene expression for each cluster. That is, within each cluster, the data at the same time point are independent replicates as suggested by the experiment design [14]; therefore, the two-sample t-test with Bonferroni correction was conducted to compare the expression levels at *t* = 1, …, *K* (*K* = 5) with that at *t* = 0, and the first time point with a *p*-value less than 0.01 was deemed as the initial change time point. Obviously, the target genes as well as TFs in the same cluster will have the same time of initial change. Then if the initial change time of a TF is later than that of its potential target genes, the corresponding TF-gene interactions are filtered out [38]. Third, we filtered out the regulatory pairs that have no any association in their expression data. For this purpose, we calculated the MIC scores (and the associated *p*-values) [41], which can measure both linear and nonlinear associations. The associations that have a *p*-value less than 0.05 were kept, and the MIC scores were assigned to the corresponding regulatory interactions as the edge weights for later use.

For further dimensional reduction, the weighted background regulatory network, which was generated at the third step in the previous paragraph, was divided into multiple smaller modules using the fast community detection algorithm [37] such that the connections within modules are much denser than those between modules [84]. That is, the nodes within each module are tightly connected by many edges, while the nodes in different modules are connected by a smaller number of edges. Therefore, the generated modules can be deemed as the functional blocks of the regularity network [85, 86]. We obtained 303 modules with the number of nodes in these modules ranging from 2 to ~200 and the number of edges ranging from 1 to ~300. Finally, it should be mentioned that the edge weights are not used in any further analysis.

### DBN and constrained LASSO

Since miRNAs repress the translation from mRNAs to proteins and TFs may regulate themselves, a directed graph model with cycles is necessarily needed to model each regulatory module. The dynamic Bayesian network (DBN) [38, 87] was considered in this study, as illustrated in Figure 5(B). Let  denote the gene expression vector of *n* genes at time *t* (*t* = 0, …, *K*), the joint distribution function can be given as follows [30]
2

where we assume that *X*^*t* + 1^ only depends on *X*^*t*^ (the Markov chain property) and the form of dependence can be described by a linear model [38, 61, 87]. More specifically, we used the model
3

where **A** is a matrix of constant regulatory coefficients, *E* is the error vector that follows a multivariate normal distribution *N*(0, *Σ*) with . In matrix **A**, let *a*_*i*,*j*_ denote the regulatory coefficient between the regulator *x*_*j*_ and the target *x*_*i*_. According to this definition, *a*_*i*,*j*_ = 0 if there exists no regulation between *x*_*j*_ and *x*_*i*_ in the background network, *a*_*i*,*j*_ > 0 when a positive regulation exists, and *a*_*i*,*j*_ < 0 when a negative regulation occurs.

To control the false positive rate in model selection for a high-dimensional linear regression problem as in Eq. (3), the LASSO method can be considered [64]. However, the existing LASSO formulations such as the adaptive LASSO [88] or the generalized LASSO [65] cannot directly accommodate the constraints posed on *a*_*i*,*j*_; for example, the regulatory coefficients associated with miRNAs are always negative. Therefore, we propose the so-called constrained LASSO, which is given as follows
4

where  denotes the coefficient vector generated by stacking the columns of **A**,  denotes the direct sum of the matrices ,…, , and  is the vector generated by stacking the expression vectors *X*^1^, …, *X*^*K*^. In addition,  is a diagonal matrix with its (*n*(*j* - 1) + *i*, *n*(*j* - 1) + *i*) entry being 1 only if *a*_*i*,*j*_ ≥ 0; otherwise, the diagonal entry is set to zero.  and  are constructed in a similar manner, representing negative and equality constraints, respectively. Also, experimentally-confirmed regulatory relationships correspond to constraints *a*_*i*,*j*_ ≠ 0; for such constraints, we introduced two nonnegative variables such that  and the constraints on  and  can be incorporated into . In addition, *λ* ≥ 0 is the penalty coefficient and the optimal *λ* value can be determined using a cross-validation procedure. It has been shown by previous studies that the optimal *λ* value should lie within the interval [0, *λ*^max^], where  [89]. We thus search the equally-spaced grids in this range using the five-fold cross validation [64] for the optimal value, which corresponds to the minimum square prediction error [64, 89]. Finally, it should be mentioned that the optimization problem in Eq. (4) is solved using quadratic programming [64, 88].

## Electronic supplementary material

Additional file 1: **Text S1, Figure TS1-TS5 and Table TS1-TS4** Construction of human regulatory background network. (DOCX 703 KB)

Additional file 2: Table S1: Details of the simulated regulatory networks with 50 (A) and 100 (B) nodes. (XLSX 18 KB)

Additional file 3: Table S2: The characterized background regulatory network (A) and the activated regulatory relationships identified by SITPR (B). (XLSX 457 KB)

Additional file 4: Table S3: The full list of the activated co-regulation motifs. (XLSX 19 KB)

Additional file 5: Table S4: The full list of enriched GO terms and documented pathways in the identified activated regulatory network during IAV infection. (XLSX 71 KB)
