# Supplementary material for: Systematic identification of transcriptional and post-transcriptional regulations in human respiratory epithelial cells during influenza A virus infection
Source: BMC Bioinformatics. 2014 Oct 4;15(1):336. doi: 10.1186/1471-2105-15-336 (PMC4287445; doi:10.1186/1471-2105-15-336)
Supplement: Supplementary file 1 — Additional file 1: Text S1, Figure TS1-TS5 and Table TS1-TS4 Construction of human regulatory background network. (DOCX 703 KB) [file 12859_2014_6643_MOESM1_ESM.docx]

Text S1. Construction of human regulatory background network

The data sources, technique steps and statistics used for building the comprehensive human background regulatory network are described here in detail. Briefly, all the databases used in the network construction are summarized in Table TS1, and the procedure of network building is shown in Figure TS1.

More specifically, we first compiled a list of human transcription factors (TFs) from FANTOM [1], UniProt [2], TRANSFAC [3] and JASPAR [4]. The human miRNAs were downloaded from miRBase [5]. The human genes and annotations were downloaded from GenBank [6] and RefSeq [7]. For consistency, TFs and genes were mapped to their corresponding NCBI symbols and Entrez gene IDs. The documented regulatory interactions between TFs and genes, such as those in TRED [8] and KEGG [9], were then extracted. Also, we incorporated the potential regulations between human TFs and genes by exploiting the documented TFBS motifs in TRANSFAC and JASPAR. Technically, we searched the promoter region of each human gene from the 5kb upstream to 1kb downstream of the transcription start site (TSS) for such motifs to determine whether a gene is the target of certain transcription factors. As illustrated in Figure TS2, the TF ‘NR2F1’ has a known TFBS ‘MA0017’, which is represented by a weighted position matrix. The sequence logo shows its nucleotide conservation. By sliding the TFBS matrix along the defined [promoter](http://en.wikipedia.org/wiki/Promoter_(genetics)) regions of human genome, the genes containing conserved putative TFBS will be identified as the targets of ‘NR2F1’. From the ENCODE project [10], we retrieved the conservation information of human TFBSs from UCSC Genome Browser [11] and Ensembl [12] databases, respectively. Specifically, UCSC’s tfbsConsSites table contains the location and score of TFBS conserved in the human/mouse/rat alignment. A binding site is considered to be conserved across the alignment if its score is above the threshold score in the species. The score and threshold are computed using the TRANSFAC matrices and the TFLOC program [11]. Similarly, Ensemble’s MotifFeatures.gff table contains the alignment information for the TFBS element matrix documented in JASPAR (by MOODS software [13]). Also, several previous studies [14, 15] have shown that there exists a strong relationship between gene co-expression/regulation and protein-protein interaction, we thus integrated human protein-protein interaction (PPI) data from HPRD [16] and KEGG as indirect regulatory relationships, which allows a more thorough and systematic exploration of the regulatory interactions [17]. That is, the TF and target proteins and TF self-regulations were incorporated into our background network.

miRNAs play a crucial role in the post-transcriptional regulation [18]. Therefore, both the documented and the potential miRNA-gene regulations are included in the human background regulatory network. Also, the interplays between TF and miRNA are considered. The experimentally-confirmed miRNA-target gene interactions were downloaded from miRTarBase [19] , TarBase [20] and miRecords [21]. Then, five widely-used databases for miRNA-target prediction were employed, including miRanda [22] , TargetScan [18], PicTar [23], MicroCosm [5] and microT [24]. Only if at least two databases contain the same predicted miRNA-target interaction, this putative post-transcriptional regulatory interaction will be included in the background network. Also, for the interplays between TFs and miRNAs, the experimentally-confirmed TF-miRNA regulations in TransmiR [25] were included. Finally, the relationships between TFs and miRNA encoding genes were identified by repeating the steps as what was done for TF-gene regulations.

For convenience, we summarized the basic information of the background network in Tables TS2 and TS3. The human background regulatory network can be downloaded from our website at http://doc.aporc.org/wiki/SITPR. Finally, the statistical measurements of the background network are presented in Fig. TS3 and Table TS4, and the SITPR pipeline is visualized in Fig. TS4. The identified 10 types of three-node network motifs in activated regulatory network are shown in Fig. TS5.

# References

1. Ravasi T, Suzuki H, Cannistraci CV, Katayama S, Bajic VB, Tan K, Akalin A, Schmeier S, Kanamori-Katayama M, Bertin N, Carninci P, Daub CO, Forrest AR, Gough J, Grimmond S, Han JH, Hashimoto T, Hide W, Hofmann O, Kamburov A, Kaur M, Kawaji H, Kubosaki A, Lassmann T, van Nimwegen E, MacPherson CR, Ogawa C, Radovanovic A, Schwartz A, Teasdale RD, *et al*: **An atlas of combinatorial transcriptional regulation in mouse and man**. *Cell* 2010, **140**(5):744-752.

2. UniProt C: **Ongoing and future developments at the Universal Protein Resource**. *Nucleic Acids Res* 2011, **39**(Database issue):D214-219.

3. Matys V, Kel-Margoulis OV, Fricke E, Liebich I, Land S, Barre-Dirrie A, Reuter I, Chekmenev D, Krull M, Hornischer K, Voss N, Stegmaier P, Lewicki-Potapov B, Saxel H, Kel AE, Wingender E: **TRANSFAC and its module TRANSCompel: transcriptional gene regulation in eukaryotes**. *Nucleic Acids Res* 2006, **34**(Database issue):D108-110.

4. Bryne JC, Valen E, Tang MH, Marstrand T, Winther O, da Piedade I, Krogh A, Lenhard B, Sandelin A: **JASPAR, the open access database of transcription factor-binding profiles: new content and tools in the 2008 update**. *Nucleic Acids Res* 2008, **36**(Database issue):D102-106.

5. Griffiths-Jones S, Saini HK, van Dongen S, Enright AJ: **miRBase: tools for microRNA genomics**. *Nucleic Acids Res* 2008, **36**(Database issue):D154-158.

6. Benson DA, Karsch-Mizrachi I, Clark K, Lipman DJ, Ostell J, Sayers EW: **GenBank**. *Nucleic Acids Res* 2012, **40**(Database issue):D48-53.

7. Pruitt KD, Tatusova T, Maglott DR: **NCBI Reference Sequence (RefSeq): a curated non-redundant sequence database of genomes, transcripts and proteins**. *Nucleic Acids Res* 2005, **33**(Database issue):D501-504.

8. Zhao F, Xuan Z, Liu L, Zhang MQ: **TRED: a Transcriptional Regulatory Element Database and a platform for in silico gene regulation studies**. *Nucleic Acids Res* 2005, **33**(Database issue):D103-107.

9. Kanehisa M, Goto S: **KEGG: kyoto encyclopedia of genes and genomes**. *Nucleic Acids Res* 2000, **28**(1):27-30.

10. Gerstein MB, Kundaje A, Hariharan M, Landt SG, Yan KK, Cheng C, Mu XJ, Khurana E, Rozowsky J, Alexander R, Min R, Alves P, Abyzov A, Addleman N, Bhardwaj N, Boyle AP, Cayting P, Charos A, Chen DZ, Cheng Y, Clarke D, Eastman C, Euskirchen G, Frietze S, Fu Y, Gertz J, Grubert F, Harmanci A, Jain P, Kasowski M*, et al*: **Architecture of the human regulatory network derived from ENCODE data**. *Nature* 2012, **489**(7414):91-100.

11. Fujita PA, Rhead B, Zweig AS, Hinrichs AS, Karolchik D, Cline MS, Goldman M, Barber GP, Clawson H, Coelho A, Diekhans M, Dreszer TR, Giardine BM, Harte RA, Hillman-Jackson J, Hsu F, Kirkup V, Kuhn RM, Learned K, Li CH, Meyer LR, Pohl A, Raney BJ, Rosenbloom KR, Smith KE, Haussler D, Kent WJ: **The UCSC Genome Browser database: update 2011**. *Nucleic Acids Res* 2011, **39**(Database issue):D876-882.

12. Flicek P, Amode MR, Barrell D, Beal K, Brent S, Carvalho-Silva D, Clapham P, Coates G, Fairley S, Fitzgerald S, Gil L, Gordon L, Hendrix M, Hourlier T, Johnson N, Kahari AK, Keefe D, Keenan S, Kinsella R, Komorowska M, Koscielny G, Kulesha E, Larsson P, Longden I, McLaren W, Muffato M, Overduin B, Pignatelli M, Pritchard B, Riat HS*, et al*: **Ensembl 2012**. *Nucleic Acids Res* 2012, **40**(Database issue):D84-90.

13. Korhonen J, Martinmaki P, Pizzi C, Rastas P, Ukkonen E: **MOODS: fast search for position weight matrix matches in DNA sequences**. *Bioinformatics* 2009, **25**(23):3181-3182.

14. Ge H, Liu Z, Church GM, Vidal M: **Correlation between transcriptome and interactome mapping data from Saccharomyces cerevisiae**. *Nat Genet* 2001, **29**(4):482-486.

15. Ravasi T, Suzuki H, Cannistraci CV, Katayama S, Bajic VB, Tan K, Akalin A, Schmeier S, Kanamori-Katayama M, Bertin N, Carninci P, Daub CO, Forrest ARR, Gough J, Grimmond S, Han J-H, Hashimoto T, Hide W, Hofmann O, Kamburov A, Kaur M, Kawaji H, Kubosaki A, Lassmann T, van Nimwegen E, MacPherson CR, Ogawa C, Radovanovic A, Schwartz A, Teasdale RD, *et al*: **An Atlas of Combinatorial Transcriptional Regulation in Mouse and Man**. *Cell*, **140**(5):744-752.

16. Peri S, Navarro JD, Kristiansen TZ, Amanchy R, Surendranath V, Muthusamy B, Gandhi TK, Chandrika KN, Deshpande N, Suresh S, Rashmi BP, Shanker K, Padma N, Niranjan V, Harsha HC, Talreja N, Vrushabendra BM, Ramya MA, Yatish AJ, Joy M, Shivashankar HN, Kavitha MP, Menezes M, Choudhury DR, Ghosh N, Saravana R, Chandran S, Mohan S, Jonnalagadda CK, Prasad CK, *et al*: **Human protein reference database as a discovery resource for proteomics**. *Nucleic Acids Res* 2004, **32**(Database issue):D497-501.

17. Cheng C, Yan K-K, Hwang W, Qian J, Bhardwaj N, Rozowsky J, Lu ZJ, Niu W, Alves P, Kato M, Snyder M, Gerstein M: **Construction and Analysis of an Integrated Regulatory Network Derived from High-Throughput Sequencing Data**. *PLoS computational biology* 2011, **7**(11):e1002190.

18. Bartel DP: **MicroRNAs: genomics, biogenesis, mechanism, and function**. *Cell* 2004, **116**(2):281-297.

19. Hsu SD, Lin FM, Wu WY, Liang C, Huang WC, Chan WL, Tsai WT, Chen GZ, Lee CJ, Chiu CM, Chien CH, Wu MC, Huang CY, Tsou AP, Huang HD: **miRTarBase: a database curates experimentally validated microRNA-target interactions**. *Nucleic Acids Res* 2011, **39**(Database issue):D163-169.

20. Sethupathy P, Corda B, Hatzigeorgiou AG: **TarBase: A comprehensive database of experimentally supported animal microRNA targets**. *Rna* 2006, **12**(2):192-197.

21. Xiao F, Zuo Z, Cai G, Kang S, Gao X, Li T: **miRecords: an integrated resource for microRNA-target interactions**. *Nucleic Acids Res* 2009, **37**(Database issue):D105-110.

22. John B, Enright AJ, Aravin A, Tuschl T, Sander C, Marks DS: **Human MicroRNA targets**. *PLoS biology* 2004, **2**(11):e363.

23. Krek A, Grun D, Poy MN, Wolf R, Rosenberg L, Epstein EJ, MacMenamin P, da Piedade I, Gunsalus KC, Stoffel M, Rajewsky N: **Combinatorial microRNA target predictions**. *Nat Genet* 2005, **37**(5):495-500.

24. Maragkakis M, Reczko M, Simossis VA, Alexiou P, Papadopoulos GL, Dalamagas T, Giannopoulos G, Goumas G, Koukis E, Kourtis K, Vergoulis T, Koziris N, Sellis T, Tsanakas P, Hatzigeorgiou AG: **DIANA-microT web server: elucidating microRNA functions through target prediction**. *Nucleic Acids Res* 2009, **37**(Web Server issue):W273-276.

25. Wang J, Lu M, Qiu C, Cui Q: **TransmiR: a transcription factor-microRNA regulation database**. *Nucleic Acids Res* 2010, **38**(Database issue):D119-122.

26. Jiang C, Xuan Z, Zhao F, Zhang MQ: **TRED: a transcriptional regulatory element database, new entries and other development**. *Nucleic Acids Res* 2007, **35**(Database issue):D137-140.

27. Mishra GR, Suresh M, Kumaran K, Kannabiran N, Suresh S, Bala P, Shivakumar K, Anuradha N, Reddy R, Raghavan TM, Menon S, Hanumanthu G, Gupta M, Upendran S, Gupta S, Mahesh M, Jacob B, Mathew P, Chatterjee P, Arun KS, Sharma S, Chandrika KN, Deshpande N, Palvankar K, Raghavnath R, Krishnakanth R, Karathia H, Rekha B, Nayak R, Vishnupriya G, *et al*: **Human protein reference database--2006 update**. *Nucleic Acids Res* 2006, **34**(Database issue):D411-414.

28. Kozomara A, Griffiths-Jones S: **miRBase: integrating microRNA annotation and deep-sequencing data**. *Nucleic Acids Res* 2011, **39**(Database issue):D152-157.

29. Lewis BP, Shih IH, Jones-Rhoades MW, Bartel DP, Burge CB: **Prediction of mammalian microRNA targets**. *Cell* 2003, **115**(7):787-798.

30. Newman MEJ: **The structure and function of complex networks**. *SIAM Review* 2003, **45**(2):167-256.

31. Barabasi AL, Albert R: **Emergence of scaling in random networks**. *Science* 1999, **286**(5439):509-512.


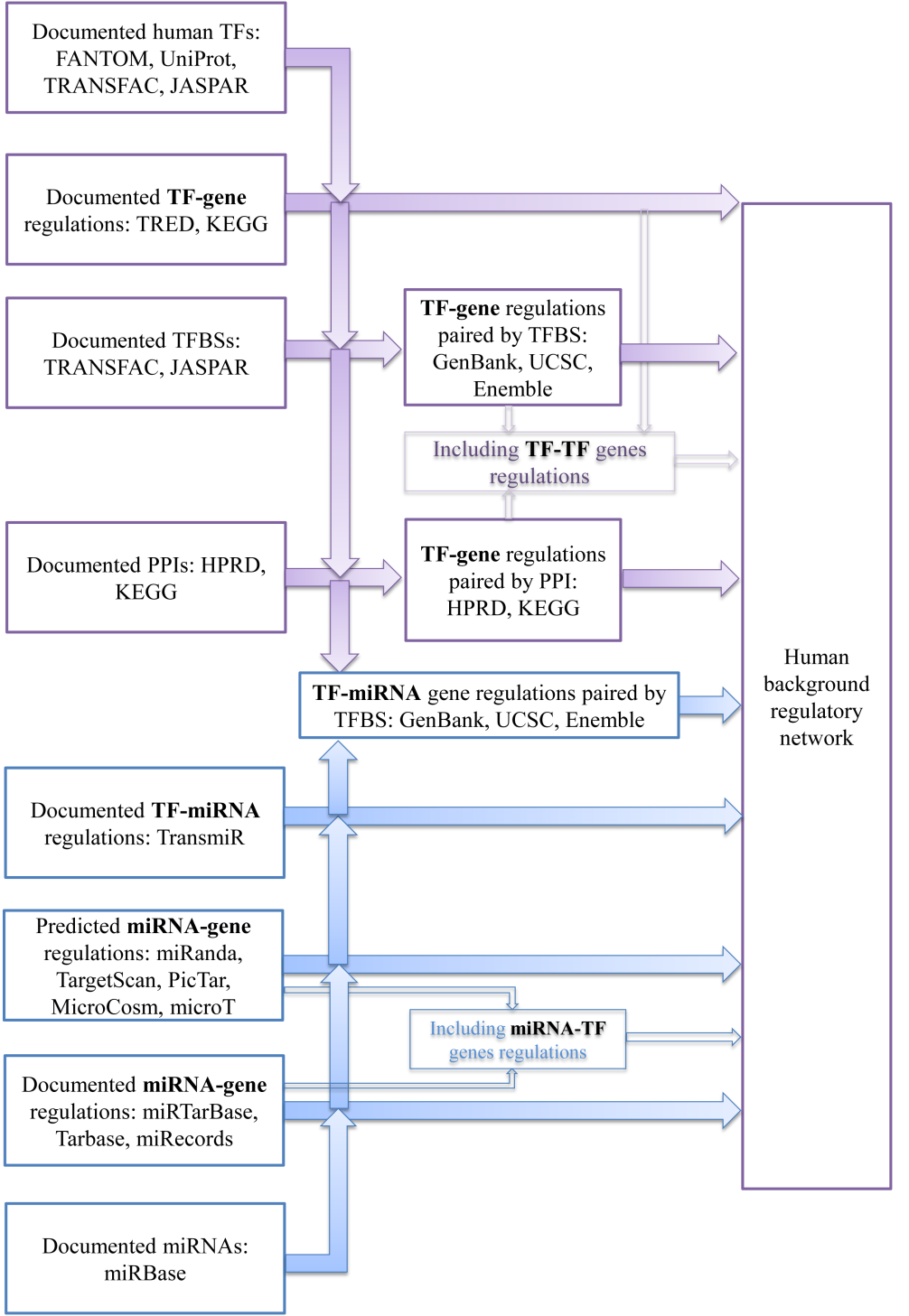


**Figure TS1: The framework of building a comprehensive regulatory network in human considering both TF and miRNA.**


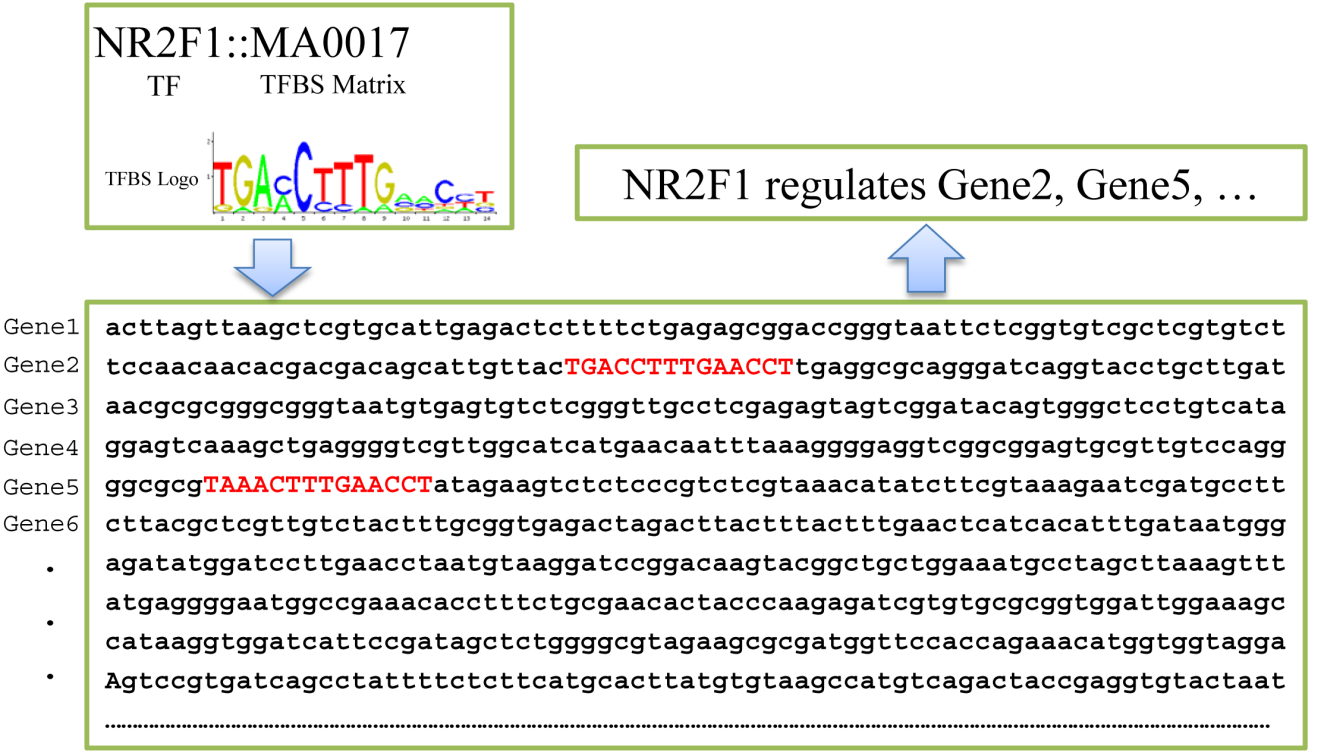


**Figure TS2. Framework of pairing TF and genes based on TFBS.**


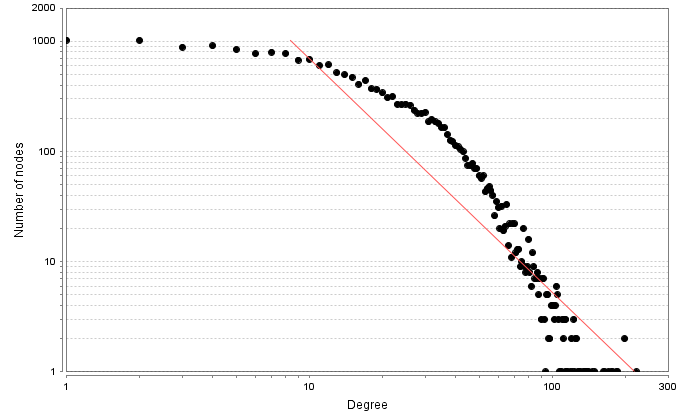


**Figure TS3. The node degree distribution of the built background regulatory network. A power law in form of $y=\alpha\cdot x^{-\gamma}$was fitted.**

**Figure TS4. The workflow of the SITPR method.**

**Figure TS5. The identified ten-types of three-node network motifs listed from ‘M1’ to ‘M10’ respectively.**

**Table TS1. Databases used to bulid the background regulatory network in human.**

| **Database** | **Description** | **Website** | **Reference** | **Version/access date** |
| --- | --- | --- | --- | --- |
| FANTOM | Functional Annotation Of Mammalian genome and is an international research consortium to assign functional annotations to the full-length complementary DNAs (cDNAs). | <http://fantom.gsc.riken.jp/> | [1] | 05-Mar-2010 |
| TRANSFAC | Transfac database is A manually curated database of eukaryotic transcription factors, their genomic binding sites and DNA binding profiles. | <http://www.gene-regulation.com/pub/databases.html> | [3] | TRANSFAC 7.0 |
| JASPAR | An open-access database of annotated, matrix-based transcription factor binding site profiles for multicellular eukaryotes. | <http://jaspar.genereg.net/> | [4] | 12-Oct-2009 |
| GenBank | A comprehensive database developed by NCBI, NIH, which contains publicly available nucleotide sequences for more than 250,00 formally described species. | <http://www.ncbi.nlm.nih.gov/genbank/> | [6] | 1-May-2012 |
| RefSeq | RefSeq provides a non-redundant collection of sequences representing genomic data, transcripts and proteins. | <http://www.ncbi.nlm.nih.gov/refseq/> | [7] | 30-May-2012 |
| UniProt | UniProt is a catalog of information on proteins and it is a central repository of protein sequence and function. | <http://www.uniprot.org/> | [2] | Release Jul-2012 |
| UCSC | The University of California, Santa Cruz Genome Browser is a database of genomic sequence and annotation data for a wide variety of organisms. | <http://genome.ucsc.edu> | [11] | hg19, GRCh37 |
| Ensembl | Ensembl is to provide a centralized resource for geneticists, molecular biologists and other researchers studying the genomes of our own species and other vertebrates and model organisms. | <http://www.ensembl.org> | [12] | Release 66 (Feb. 2012) |
| TRED | Transcriptional Regulatory Element Database (TRED) is an integrated repository repository for both cis- and trans- regulatory elements in mammals. It contains the curated regulations between TF and target gene. | <http://rulai.cshl.edu/TRED/> | [26] | 12-Feb-2012 |
| KEGG | KEGG is a widely used pathway database resource for understanding high-level linkage functions and utilities of biological system. | <http://www.genome.jp/kegg/> | [9] | 03-Dec-2010 |
| HPRD | HPRD is a curated human protein-protein interaction database. | <http://www.hprd.org> | [27] | Release 9 |
| miRBase | miRBase database is a searchable database of published miRNA sequences and annotation. | <http://www.mirbase.org/> | [28] | Release 18 |
| TransmiR | TransmiR is a transcription factor-microRNA regulation database | <http://202.38.126.151/hmdd/mirna/tf/> | [25] | Version 1.2 |
| miRanda | miRanda is a miRNA target prediction method based on dynamic programming algorithm | <http://www.microrna.org> | [22] | Release August 2010 |
| TargetScan | TargetScan is an algorithm to predict biological targets of miRNAs by searching for the presence of conserved 8mer and 7mer sites that match the seed region of each miRNA. | <http://www.targetscan.org/> | [29] | Release 5.0 |
| PicTar | PicTar is a computational method for identifying common targets of microRNAs. | <http://pictar.mdc-berlin.de/> | [23] | 26-Mar-2007 |
| MicroCosm | MicroCosm Targets (formerly miRBase Targets) is a web resource containing computationally predicted targets for microRNAs across many species. | <http://www.ebi.ac.uk/enright-srv/microcosm/htdocs/targets/v5/> | [5] | Version v5 |
| MicroT | DIANA-microT is a combined computational-experimental approach predicts human microRNA targets. | <http://www.microrna.gr/microT> | [24] | Version v3.0 |
| miRTarBase | miRTarBase is a database which curates experimentally validated microRNA-target interactions. | <http://miRTarBase.mbc.nctu.edu.tw/> | [19] | Release 2.5 (Oct-2011) |
| Tarbase | Tarbase collectes available miRNA targets derived from all contemporary experimental techniques (gene specific and high-throughput). | <http://www.microrna.gr/tarbase> | [20] | Version 5.0 |
| miRecords | miRecords is a resource for animal miRNA-target interactions. The validated targets component is used, which is a large, high-quality database of experimentally validated miRNA targets. | <http://miRecords.umn.edu/miRecords> | [21] | 25-Nov-2010 |

**Table TS2. Summary of the original background regulatory network in human.**

| **Element** | **Description** | **Number** |
| --- | --- | --- |
| Node | All the TFs, miRNAs and target genes | 23,079 |
| Edge | All the regulatory relationships | 369,277 |
| TF | The documented transcription factors | 1,456 |
| miRNA | The documented microRNAs | 1,904 |
| Gene | The target genes | 19,719 |
| TF-gene | The TF-target gene regulations | 149,841 |
| TF-TF | The TF-TF gene self-regulations | 361 |
| TF-miRNA | The TF-miRNA gene regulations | 21,744 |
| miRNA-gene | The miRNA-target gene regulations | 171,477 |
| miRNA-TF | The miRNA-TF gene regulations | 25,854 |

**Table TS3: Summary of the background regulatory network in human after incorporating the mRNA and miRNA expression data (GSE36553 and GSE36461, respectively).**

| **Element** | **Description** | **Number** |
| --- | --- | --- |
| Node | All the TFs, miRNAs and target genes | 18,964 |
| Edge | All the regulatory relationships | 335,963 |
| TF | The documented transcription factors | 1,441 |
| miRNA | The documented microRNAs | 881 |
| Gene | The target genes | 16,642 |
| TF-gene | The TF-target gene regulations | 132,607 |
| TF-TF | The TF-TF gene self-regulations | 359 |
| TF-miRNA | The TF-miRNA gene regulations | 10,302 |
| miRNA-gene | The miRNA-target gene regulations | 167,387 |
| miRNA-TF | The miRNA-TF gene regulations | 25,308 |

**Table TS4. The statistical measurements of the background network. The parameter definitions can be found in [30, 31].**

| Clustering coefficient | 0.117 | Shortest paths | 34,134,823 |
| --- | --- | --- | --- |
| Connected components | 3 | Characteristic path length | 3.171 |
| Network diameter | 8 | Average number of neighbors | 34.869 |
